# Association of Irisin/FNDC5 with ERRα and PGC-1α Expression in NSCLC

**DOI:** 10.3390/ijms232214204

**Published:** 2022-11-17

**Authors:** Katarzyna Nowińska, Karolina Jabłońska, Urszula Ciesielska, Aleksandra Piotrowska, Katarzyna Haczkiewicz-Leśniak, Konrad Pawełczyk, Marzenna Podhorska-Okołów, Piotr Dzięgiel

**Affiliations:** 1 Division of Histology and Embryology, Department of Human Morphology and Embryology, Wroclaw Medical University, 50-368 Wroclaw, Poland; 2 Division of Ultrastructural Research, Wroclaw Medical University, 50-368 Wroclaw, Poland; 3 Lower Silesian Centre of Oncology, Pulmonology and Haematology, 53-439 Wroclaw, Poland; 4 Department of Physiotherapy, Wroclaw University School of Physical Education, 51-612 Wroclaw, Poland

**Keywords:** irisin, *FNDC5*, ERRα, *ESRRA*, PGC-1α, NSCLC, non-small cell lung cancer

## Abstract

The rapid growth and division of cancer cells are associated with mitochondrial biogenesis or switching to glycolysis. ERRα, PGC-1α and irisin/FNDC5 are some of the proteins that can influence these processes. The aim of this study was to determine the correlation of these proteins in non-small cell lung cancer (NSCLC) and to investigate their association with clinicopathological parameters. Immunohistochemistry reactions were performed on tissue microarrays (860 NSCLC, 140 non-malignant lung tissue). The normal fibroblast cell line (IMR-90) and lung cancer cell lines (NCI-H1703 and NCI-H522) were used as co-cultures. The mRNA levels of *FNDC5* and *ESRRA* (encoding ERRα) were assessed in IMR-90 cells after co-culture with lung cancer cells. We observed a decreased level of ERRα with an increase in tumor size (T), stages of the disease, and lymph node metastases (N). In the adenocarcinoma (AC) subtype, patients with a higher ERRα expression had significantly longer overall survival. A moderate positive correlation was observed between *FNDC5* mRNA and *ESRRA* mRNA in NSCLCs. The expression of *FNDC5* mRNA in IMR-90 cells increased after 24 h, and *ESRRA* gene expression increased after 48 h of co-culture. The ERRα receptor with PGC-1α participates in the control of FNDC5/irisin expression. Normal fibroblasts revealed an upregulation of the *FNDC5* and *ESRRA* genes under the influence of lung cancer cells.

## 1. Introduction

Over the past 10 years, the treatment of lung cancer, especially of non-small cell lung cancer (NSCLC), has changed considerably. A better understanding of tumor biology has enabled the development of targeted therapies, which has opened the way for personalized medicine. Most treatments available today are selected based on changes in lung cancer cells such as EGFR, KRAS, or ALK mutations [1]. In recent years, increasing attention has been paid to the cross-talk and interaction between cancer cells and the tumor microenvironment (TME). According to current knowledge, tumors are heterogeneous masses of cells. The TME consists of various cell populations, termed stromal cells, in a complex matrix. Many cell types coexist with transformed cancer cells. These coexisting cell types include fibroblasts, endothelial and immune cells. As the tumor grows, these cells transform into cancer-associated fibroblasts (CAFs), tumor-associated dendritic cells (TADCs), tumor-associated neutrophils (TANs), tumor-associated macrophages (TAMs) and regulatory T-lymphocytes. Based on these studies, immunotherapy using anti-PD-L1 and PD-1 antibodies can be applied [2,3,4]. Increasing knowledge of the relationship between cancer cells and the stroma, and understanding of the changes occurring in the tumor environment, may affect the subsequent invention of targeted therapy.

Alterations in the metabolism of cancer cells are critical and essential for the maintenance of carcinogenesis. Recent studies indicate a dynamic relationship between glycolysis, mitochondrial functioning and synthesis pathways in neoplastic cells [5,6]. The rapid growth and division of neoplastic cells are associated with energy requirements that can be provided by mitochondrial biogenesis or by switching to glycolysis. Mitochondrial biogenesis occurs in response to the high energy requirements of the cell. ERRα, PGC1α and irisin/FNDC5 are the proteins that can influence these processes [5,7]. In many studies, an increased level of the expression of these proteins was observed in neoplastic cells [5,8]. The most characteristic alteration in the metabolism of cancer cells is their ability for glucose uptake generated by anaerobic glycolysis. Switching to anaerobic glycolysis results in increased cell proliferation [4]. Glucose synthesis occurs as a result of glycolysis, despite the presence of oxygen. Alterations in the metabolism of cancer cells are necessary and crucial to maintain carcinogenesis. It is believed that CAFs in the TME may alter the metabolism of neoplastic cells and could promote cancer development. CAFs can influence cancer progression by secreting growth factors and various cytokines. These factors affect the proliferation and migration of cancer cells, and also stimulate angiogenesis in tumors [9]. However, the mechanism has not been confirmed or clarified yet. Our previous study [10] also indicated the relationship between irisin expression in stromal CAFs and the survival of patients with lung cancer. Our previous studies showed the implication of irisin/FNDC5 expression in tumor cells and stromal cells in progression of NSCLC [8,10]. The relationship between irisin and ERRα or PGC-1α has not been studied in any of the cancers, particularly in NSCLC.

Irisin is a protein expressed in normal cells, and its highest level is observed in cells with a high metabolism, such as fibers of skeletal muscle, cardiomyocytes, adipocytes and hepatocytes [7,8,11,12]. The protein has also been detected in cancer cells, including cancers of the digestive system, breast, ovary, lungs, and larynx [10,13,14,15,16,17,18,19]. Our previous studies on the determination of the location and the level of expression of irisin in NSCLC revealed that it was present both in the cytoplasm of cancer cells and in tumor stromal fibroblasts [10]. These cells are also characterized by increased metabolism. High irisin expression found in the lung tumor stroma was associated with shorter survival times [10]. Irisin shows many pleiotropic effects on tissues and signaling pathways [20]. It is related to glucose homeostasis and increases glucose uptake by activating the AMPKα2 kinase and p38 MAPK-dependent kinase in muscle cells [21].

Irisin is encoded by the *FNDC5* gene, whose expression is controlled by the peroxisome proliferator activated receptor gamma coactivator 1 alpha (PGC-1α). PGC-1α is a transcriptional co-activator which does not bind directly to DNA. Studies indicated that the estrogen-related receptor alpha (ERRα) could be a factor that plays a role in PGC-1α binding to DNA [5,8,22,23,24,25]. ERRα is encoded by the *ESRRA* gene and is an orphan nuclear receptor, which has two domains. One of them allows the interaction with DNA, and the second one with a ligand. The ERRα structure is similar to that of the estrogen receptor alpha (ERα), but this receptor does not bind to natural estrogens [24,25,26,27]. ERRα interacts with a canonical sequence of the estrogen response elements (ERRE). ERRα and ERα could compete with each other to bind to similar DNA elements. Together, ERRα binds to DNA, in complex with PGC-1α, to regulate the activity of genes such as *FNDC5*. The murine *Fndc5* gene and its promoter were investigated for ERRα binding sites (ERREs). Their study indicated two putative ERREs. One of them was located upstream of the transcriptional start site, while the other was located in the fourth *Fndc5* gene intron. It is believed that PGC-1α/ERRα cooperates with certain oncogenes to reprogram the metabolism of neoplastic cells. This suggests that there is a positive relationship between PGC-1α and ERRα expression levels in tissues [5,8,22,23,24,25]. The ERRα binds to a variety of response gene elements or gene promoters, including osteopontin [26], lactoferrin and medium-chain acyl coenzyme A dehydrogenase (MCAD), or thyroid hormone receptor gene promoters. The ERRα causes their activation [25]. Studies indicated that cAMP increased phosphorylation of ERRα and promoted its transport to the nucleus. Additionally, this orphan nuclear receptor can be acetylated or sumoylated. Posttranslational modifications may alter the activity of the ERRα and its regulatory properties [28]. The ERRα maintains the glucose and lipid metabolism homeostasis, bone metabolism homeostasis, oxidation and oxidative phosphorylation of fatty acids and mitochondrial biogenesis. Additionally, the expression of this receptor and the expression of irisin were found in metabolically active tissues such as kidney, heart, brain, intestine, liver, brown adipose and skeletal muscle. These tissues utilize fatty acids for energy production [8,24,26,27]. Its increased expression was observed in breast cancer. High ERRα expression was associated with the induction of epithelial-mesenchymal transition (EMT) in breast cancer cells and a worse prognosis for patients [29]. Li et al. [30] showed that increased ERRα expression was associated with shorter survival rates in patients with pulmonary adenocarcinoma (AC). Apart from the influence of irisin on cell proliferation and migration, it is mainly associated with metabolic changes in adipocytes [31]. Additionally, the relationship of the transcription factor PGC-1α and the ERRα receptor with the FNDC5 expression prompted us to conduct the studies described in this paper [5,23,30,32,33].

The relationship of irisin expression with the levels of ERRα and PGC-1α expression in lung cancer has not been investigated yet. The aim of the study was to determine the relationship between irisin expression in cancer cells and stromal cells of NSCLC with the ERRα receptor and PGC-1α. Additionally, our research determined the level of irisin protein and ERRα expression in the receptor, as well as the mRNA level of the *FNDC5* and *ESRRA* genes in the tissues of NSCLC and normal tissues. Confirmation of a relationship between these proteins could suggest that irisin is involved in reprogramming the metabolism of lung cancer cells. In addition, we investigated the relationship of ERRα and PGC-1α with diagnostic markers differentiating NSCLC subtypes such as p63 and TTF-1. Moreover, the association of ERRα and PGC-1α with the Ki-67 antigen, which is a recognized marker of cell proliferation, was verified [10]. The correlation of ERRα and PGC-1α with PD-L1, which is used to assess the validity of immunotherapy, was investigated [2]. The relationship of ERRα and PGC-1α with EGFR, which is associated with molecularly targeted therapy, was checked [34]. However, there have been no in vitro studies to determine the impact of lung cancer cells on changes in the level of *FNDC5* or *ESRRA* expression in lung fibroblasts. Changes in the expression level of the *FNDC5* and *ESRRA* genes in fibroblasts simulating the lung tumor stroma after incubation with lung cancer cells have been described for the first time. Moreover, the aim of the study was to compare the expression level of irisin, ERRα and PGC-1α with clinicopathological factors. The investigation of the association between these two proteins has not been performed yet on such a large study group of 860 NSCLC tumors.

## 2. Results

### 2.1. Immunohistochemical (IHC) Detection of Irisin, ERRα and PGC-1α Expression in Tissue Microarrays (TMA) with NSCLC

**Figure 1:**
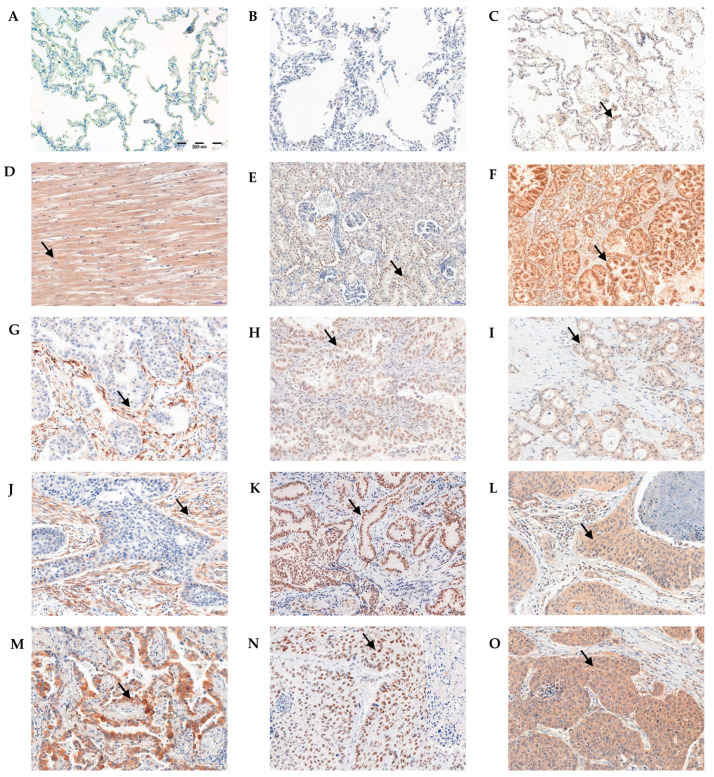
Immunohistochemical reactions (IHC) indicating lack of irisin (**A**) and ERRα (**B**) expression. Weak positive IHC reaction for PGC-1α (**C**) in non-malignant lung tissue (NMLT). Positive cytoplasmic IHC reaction indicating irisin expression in skeletal muscle (positive control—**D**). Irisin expression in NSCLC cancer cells and stromal cells (grade of malignancy G1—**G**, G2—**J**, G3—**M**). Nuclear expression of ERRα in kidney (positive control—**E**). ERRα expression in NSCLC cancer cells (G1—**H**, G2—**K**, G3—**N**). Positive cytoplasmic expression of PGC-1α in prostate (positive control—**F**). PGC-1α in NSCLC cancer cells and stromal cells (G1—**I**, G2—**L**, G3—**O**). Magnification ×200. Arrows indicate a positive reaction.

The slides stained with IHC underwent pathomorphological examination. The assessment of IHC reactions was conducted by two independent pathologists. Irisin was observed in the cytoplasm of tumor cells and tumor stromal fibroblasts in NSCLC (Figure 1G,J,M). On the other hand, ERRα was present in the nuclei of NSCLC cells (Figure 1H,K,N). We also noticed the expression of the transcription factor PGC-1α in cancer cells and tumor stromal cells (Figure 1I,L,O). We did not observe the expression of ERRα or irisin in 140 non-malignant lung tissues (NMLT) (Figure 1A,B). The PGC-1α expression was absent or weak in NMLTs.

### 2.2. Association of Irisin/FNDC5 with Clinicopathological Parameters of NSCLC

**Figure 2:**
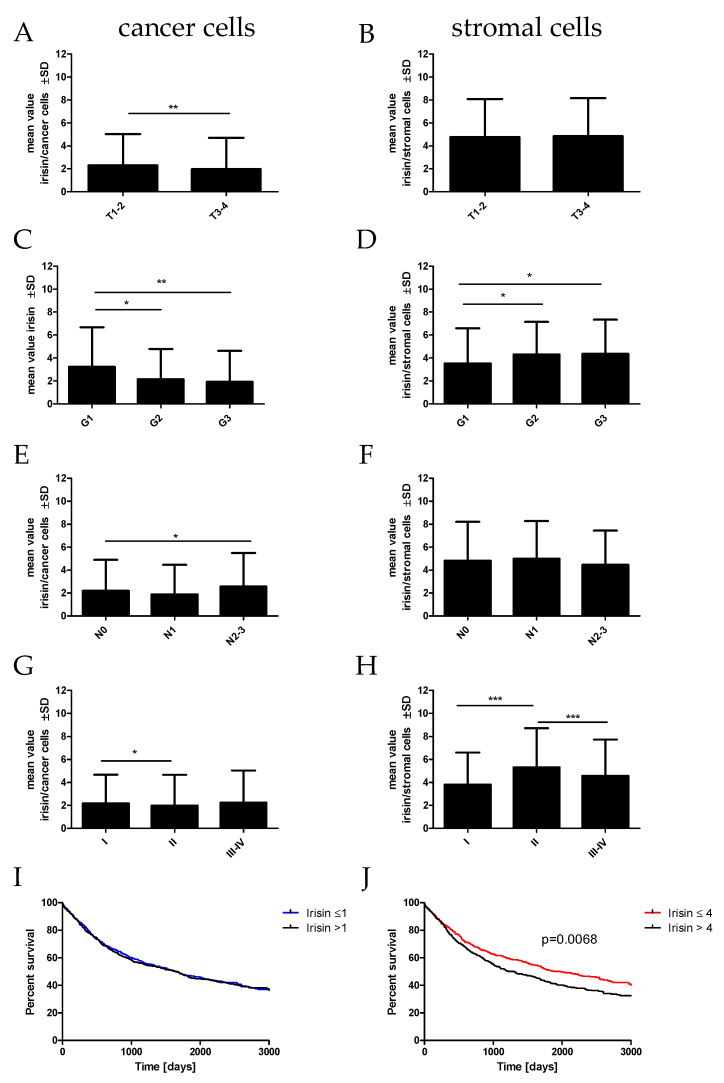
Comparison of irisin expression levels detected by immunohistochemistry (IHC) in NSCLC (n = 860) cells (**A**,**C**,**E**,**G**) and in stromal cells (**B**,**D**,**F**,**H**) according to the tumor size (**A**,**B**), malignancy grade (**C**,**D**), lymph node status (**E**,**F**), and tumor stage (**G**,**H**), * *p* ≤ 0.05, ** *p* ≤ 0.005, *** *p* ≤ 0.001. Kaplan–Meier survival curves show the prognostic impact of irisin expression levels in cancer cells (**I**) and stromal cells (**J**) on overall survival (OS) in patients with NSCLC. Patients were grouped according to the median value of expression levels.

The statistical analyses of the association of irisin/FNDC5 expressed in cancer cells in NSCLC with clinical and pathological factors were performed (Table 1, Figure 2). We noticed a significantly higher level of irisin expressed in cancer cells in T1-T2 (*p* = 0.0055; mean 2.29 ± 2.6 SD) compared to T3-T4 (mean 1.97 ± 1.5 SD). We observed statistically significant differences in irisin expression in higher grades of malignancy (G) (Kruskal–Wallis test, *p* = 0.0090). Irisin expression was highest in G1 (mean 3.23 ± 3.4 SD) and decreased significantly in G2 (mean 2.14 ± 2.6 SD; G1 vs. G2 *p* = 0.0213) and G3 (mean 1.93 ± 2.7 SD; G1 vs. G3 *p* = 0.0034). Moreover, the expression of irisin increased in the SI compared to the SII stage of the disease (*p* = 0.0141; SI—mean 2.45 ± 1.3 SD, SII—mean 2.33 ± 1.3 SD). Additionally, we observed a significant increase in irisin expression in the group of patients with lymph node metastases (N1) (mean 1.86 ± 2.6 SD) in comparison with the group with mediastinal node metastases (N2) (mean 2.57 ± 2.8 SD; N1 vs. N2 *p* = 0.0200).

Statistical analyses of the irisin/FNDC5 expression levels in stromal cells in NSCLC with clinical and pathological factors were also performed (Table 1, Figure 2). Irisin expression in stromal cells decreased in higher G (G1 mean 3.53 ± 3.0, G2 mean 4.30 ± 2.7 SD; G3 mean 4.35 ± 2.9; G1 vs. G2 *p* = 0.0381, G1 vs. G3 *p* = 0.0362). Moreover, the expression of irisin in stromal cells was lower in the SI than the SII stage of the disease (*p* = 0.0002; SI—mean 3.82 ± 2.8 SD, SII—mean 5.33 ± 3.3 SD). However, the level of irisin expression in SII was higher than in SIII-IV (*p* = 0.0075; SIII-IV mean 4.57± 3.1). We also found that patients with higher irisin expression in stromal cells had significantly shorter overall survival (OS) (*p* = 0.0068) (Figure 2J).

### 2.3. Association of ERRα with Clinicopathological Parameters of NSCLC

**Figure 3:**
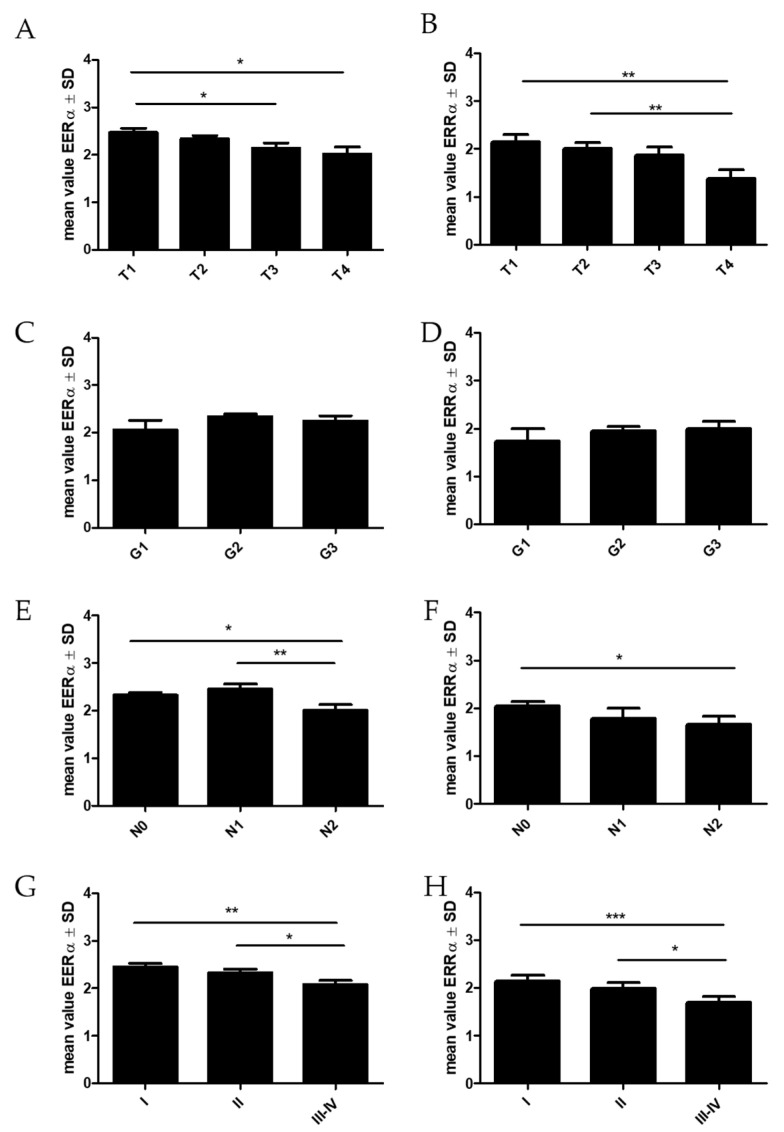
Comparison of ERRα receptor expression levels detected by immunohistochemistry (IHC) in non-small cell lung cancer NSCLC (n = 860, **A**,**C**,**E**,**G**) and adenocarcinoma subtype—AC (n = 344, **B**,**D**,**F**,**H**) according to the tumor size (**A**,**B**), the grade of malignancy (**C**,**D**), the lymph node status (**E**,**F**), and the tumor stage (**G**,**H**), * *p* ≤ 0.05, ** *p* ≤ 0.005, *** *p* ≤ 0.001.

Statistical analyses of the relationship of ERRα with clinical and pathological factors in NSCLC and its subtypes were also performed (Table 2, Figure 3). We observed a decreased level of ERRα expression with an increase in T (*p* = 0.0335). We noticed a significantly higher level in T1 (*p* = 0.0114; mean 2.46 ± 1.3 SD) compared to T4 (mean 2.02 ± 1.4 SD) and T3 (*p* = 0.0312; mean 2.15 ± 1.4 SD). We also noticed statistically significant differences in ERRα expression between the groups of patients with or without lymph node metastases (Kruskal–Wallis test, *p* = 0.0214). ERRα expression was highest in N1 (mean 2.45 ± 1.3 SD) and decreased significantly in N2 (mean 2.00 ± 1.4 SD N1 vs. N2 *p* = 0.0092; N0 vs. N2 *p* = 0.0175). Moreover, the expression of ERRα decreased in the subsequent stages of the disease (Kruskal–Wallis test, *p* = 0.0047; SI—mean 2.45 ± 1.3 SD, SII—mean 2.33 ± 1.3 SD, SIII—mean 2.07 ± 1.4 SD).

**Figure 4:**
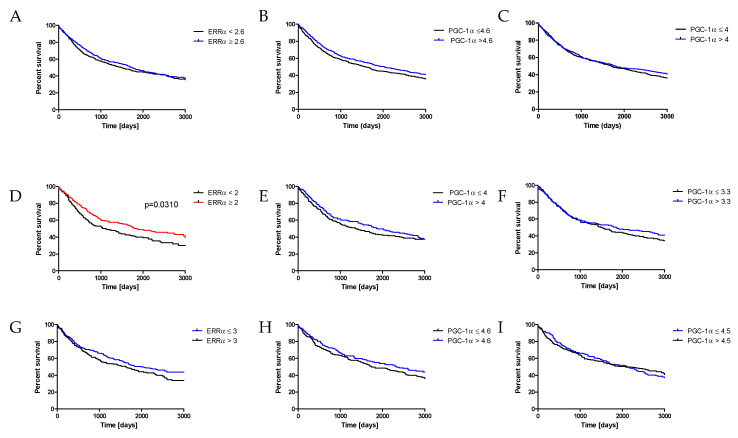
Kaplan–Meier survival curves show the prognostic impact of ERRα expression levels on overall survival (OS) in patients with NSCLC (**A**), AC (**D**), SCC (**G**). Kaplan–Meier survival curves show the prognostic impact of PGC-1α expression levels in cancer cells on overall survival (OS) in patients with NSCLC (**B**), AC (**E**), SCC (**H**). Kaplan–Meier survival curves show the prognostic impact of PGC-1α expression levels in stromal cells on overall survival (OS) in patients with NSCLC (**C**), AC (**F**), SCC (**I**). Patients were grouped according to the median value of expression levels.

In the adenocarcinoma (AC) subtype, we observed similar tendencies to those in NSCLC. ERRα expression decreased in higher T (T1 vs. T4 *p* = 0.0061; T2 vs. T4 *p* = 0.0097) and N stages (N0 vs. N2 *p* = 0.0494). In this subtype of lung cancer, we also found that patients with higher ERRα expression had significantly longer OS times (*p* = 0.0310) (Figure 4).

### 2.4. Association of PGC-1α with Clinicopathological Parameters of NSCLC

**Figure 5:**
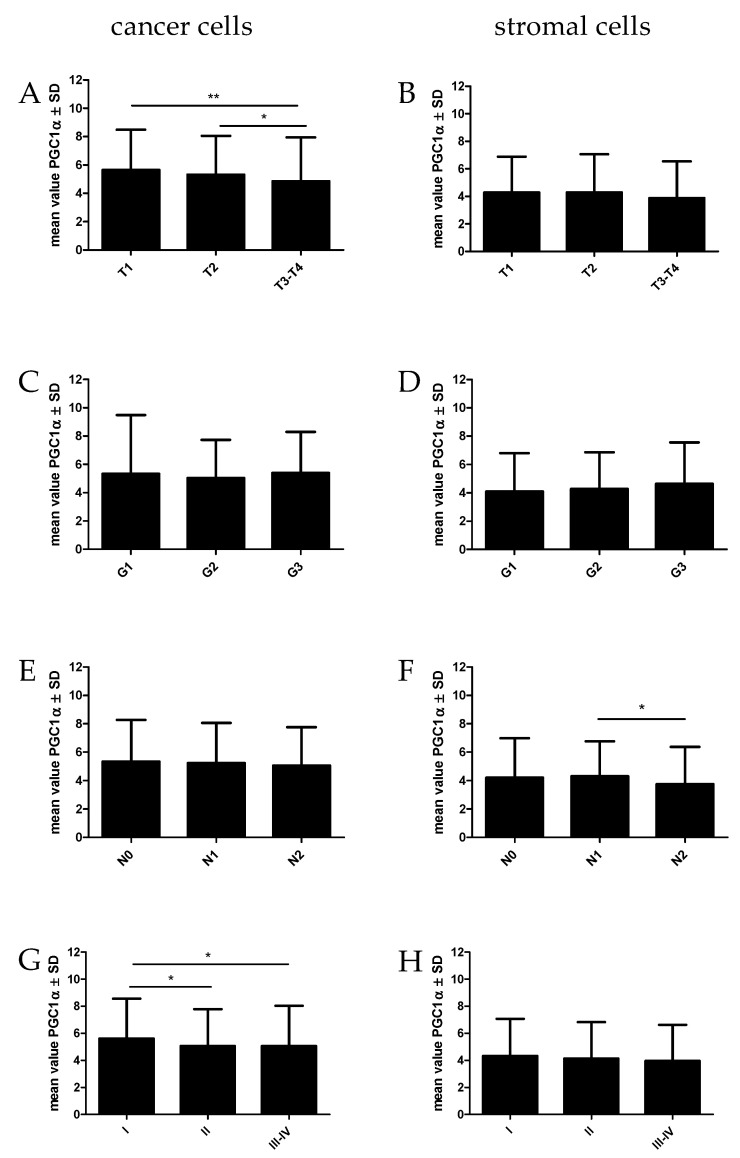
Comparison of PGC-1α expression levels detected by immunohistochemistry (IHC) in NSCLC (n = 860) cells (**A**,**C**,**E**,**G**) and in stromal cells (**B**,**D**,**F**,**H**) according to the tumor size (**A**,**B**), malignancy grade (**C**,**D**), lymph node status (**E**,**F**), and tumor stage (**G**,**H**). * *p* ≤ 0.05, ** *p* ≤ 0.005.

The statistical analyses of the relationship between PGC1α found in cancer cells and stromal cells with clinical and pathological factors in NSCLC are given in Figure 5 and Table 3. We observed a decreased level of PGC-1α expression in cancer cells with an increase in tumor size (T) (Kruskal–Wallis test, *p* = 0.0064). Significant differences were found between T1 vs. T3-T4 (*p* = 0.0027) and T2 vs. T3-T4 (*p* = 0.0144). Moreover, PGC1α expressed in cancer cells decreased in advanced stages of the disease (Kruskal–Wallis test, *p* = 0.0339). PGC-1α expression levels significantly decreased in SII vs. SI (*p* = 0.0482) and in SIII-IV vs. SI (*p* = 0.0173).

PGC-1α expression was noted in stromal cells in NSCLC tumors. We observed similar trends in the levels of PGC-1α expression in stromal cells to those when the transcription factor was observed in cancer cells. PGC-1α expression levels were lower in higher tumor size and advanced stages in NSCLC patients. However, we noticed a lower level of PGC-1α expression in the group of patients with mediastinal node metastases (N2) than in the group with N1 (*p* = 0.0339). We did not observe the association of PGC-1α expression levels with OS in NSCLC patients.

### 2.5. The Association of ERRα and PGC-1α with Diagnostic Markers in NSCLC (TTF-1, p63, Ki-67, EGFR and PD-L1)

**Figure 6:**
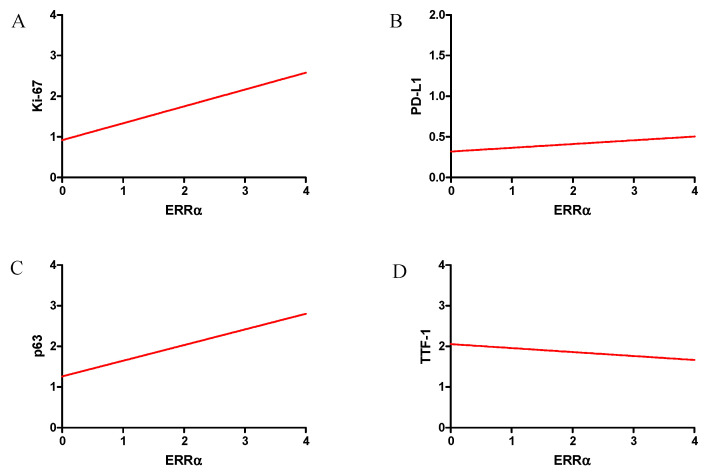
Correlations of ERRα receptor expression levels with diagnostic markers were strong positive—Ki-67 (**A**), moderate positive—EGFR (**B**), moderate positive—p63 (**C**) and weak positive—PD-L1 (**D**) in NSCLC (n = 860).

In our study, the correlations of ERRα with important diagnostic markers in NSCLC were investigated. The graphs presenting the ERRα correlation with the examined markers are shown in Figure 6. We observed a high positive correlation with the Ki-67 proliferation antigen (r = 0.47, *p* < 0.0001). Moreover, a mean positive correlation was also observed for the status of EGFR receptors (*p* = 0.31, *p* < 0.0001) and the expression of p63 protein (r = 0.32, *p* < 0.0001). We found a weak positive correlation between ERRα and the PD-L1 status (r = 0.10, *p* = 0.0041) and a weak negative correlation with TTF-1 (r = 0.01, *p* = 0.0199).

**Figure 7:**
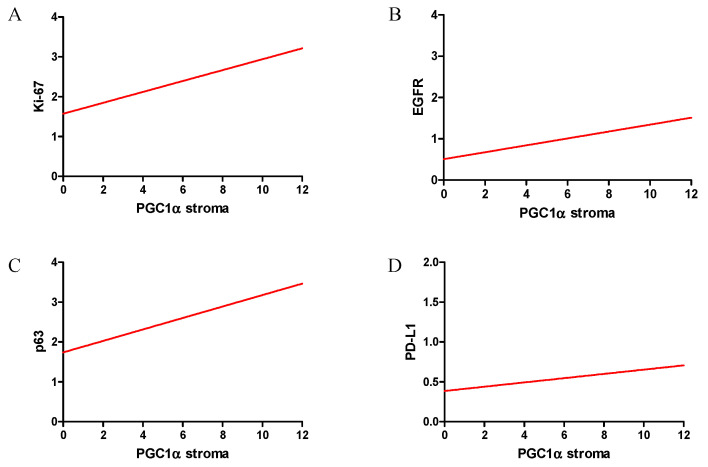
Correlations of PGC-1α expression levels in stromal cells with diagnostic markers were moderate positive—Ki-67 (**A**), weak positive—EGFR (**B**), weak positive—p63 (**C**) and weak positive—PD-L1 (**D**) in NSCLC (n = 860).

In our study, we also analyzed the relationship between PGC-1α expression and diagnostic markers in NSCLC (Figure 7). We found a mean positive correlation with the Ki-67 antigen (r = 0.30, *p* < 0.0001), as well as a weak correlation with the status of EGFR receptors (r = 0.21, *p* < 0.0001), PD-L1 (r = 0.14, *p* = 0.0002) and p63 (r = 0.22, *p* < 0.0001). We did not observe any correlation between PGC-1α and TTF-1.

### 2.6. Correlations between Irisin/FNDC5, PGC-1α and ERRα

**Figure 8:**
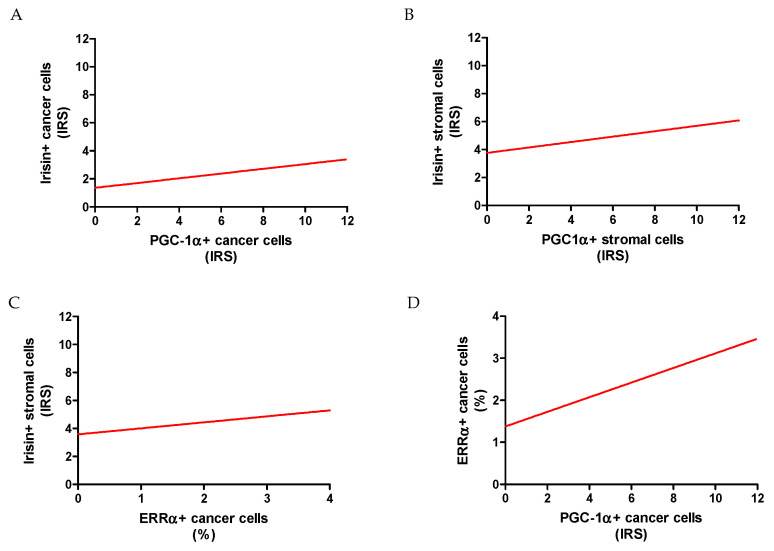
Correlations between irisin expressed in cancer cells with PGC-1α expressed in cancer cells (**A**) and stromal cells with PGC-1α (stromal cells) (**B**), and ERRα receptor (**C**). Correlations between ERRα and PGC-1α in NSCLC (n = 860) cancer cells (**D**).

We found a moderate positive correlation between ERRα and PGC-1α (r = 0.37, *p* < 0.0001). Additionally, we noticed an association between PGC-1α expressed in the stroma and in cancer cells (r = 0.20, *p* < 0.0001). A weak positive correlation was also found between PGC-1α and irisin protein expression in the tumor stroma (r = 0.23, *p* < 0.0001) (Figure 8). Irisin expression in cancer cells correlated poorly with PGC1α levels in the same cells (r = 0.19, *p* < 0.0001).

Statistical analysis was also performed to check the relationship between the level of irisin expression and ERRα. In NSCLC, a weak positive correlation was observed between irisin expression in the tumor stroma and ERRα (r = 0.23, *p* < 0.0001). However, we did not observe any correlation between ERRα and irisin expression in lung cancer cells. In the AC subtype, irisin in the stroma correlated weakly positively with the expression of the ERRα receptor (r = 0.15; *p* = 0.0046). In the AC, we also observed a correlation between irisin in lung cancer cells and ERRα expression (r = 0.14; *p* = 0.0067).

### 2.7. Comparison between mRNA FNDC5 and mRNA ESRRA Expression Levels in NSCLCs

**Figure 9:**
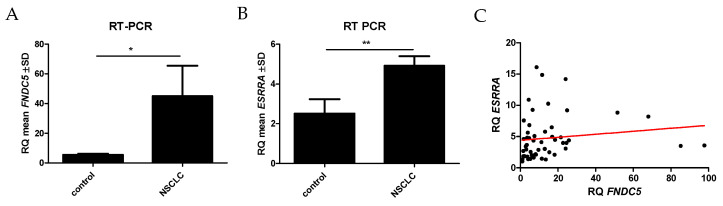
Comparison between control (n = 16) and NSCLCs (n = 56) of *FNDC5* mRNA (**A**) and *ESRRA* mRNA (**B**) expression levels. The moderate positive correlation between mRNA *FNDC5* and mRNA *ESRRA* expression levels in NSCLC patients (**C**), * *p* ≤ 0.05, ** *p* ≤ 0.005.

The levels of *FNDC5* mRNA (encoding irisin) and *ESRRA* mRNA (encoding ERRα receptor) expression were evaluated and compared in NSCLC and normal lung tissues. *FNDC5* gene expression levels were significantly higher in NSCLCs (mean RQ 44.9 ± 153.6 SD) in comparison to NMLTs (mean RQ 5.5 ± 3.1 SD; *p* = 0.0159) (Figure 9A). Similarly, *ESRRA* mRNA expression levels were significantly higher in NSCLCs (mean RQ 4.9 ± 3.6 SD) than in NMLT expression levels (mean RQ 2.5 ± 0.6 SD; *p* = 0.0070) (Figure 8B). We found a moderate positive correlation between *FNDC5* mRNA and *ESRRA* mRNA (r = 0.32, *p* = 0.0180) (Figure 9C).

### 2.8. Influence of Lung Cancer Cells on FNDC5 mRNA Expression Levels in an In Vitro Model

**Figure 10:**
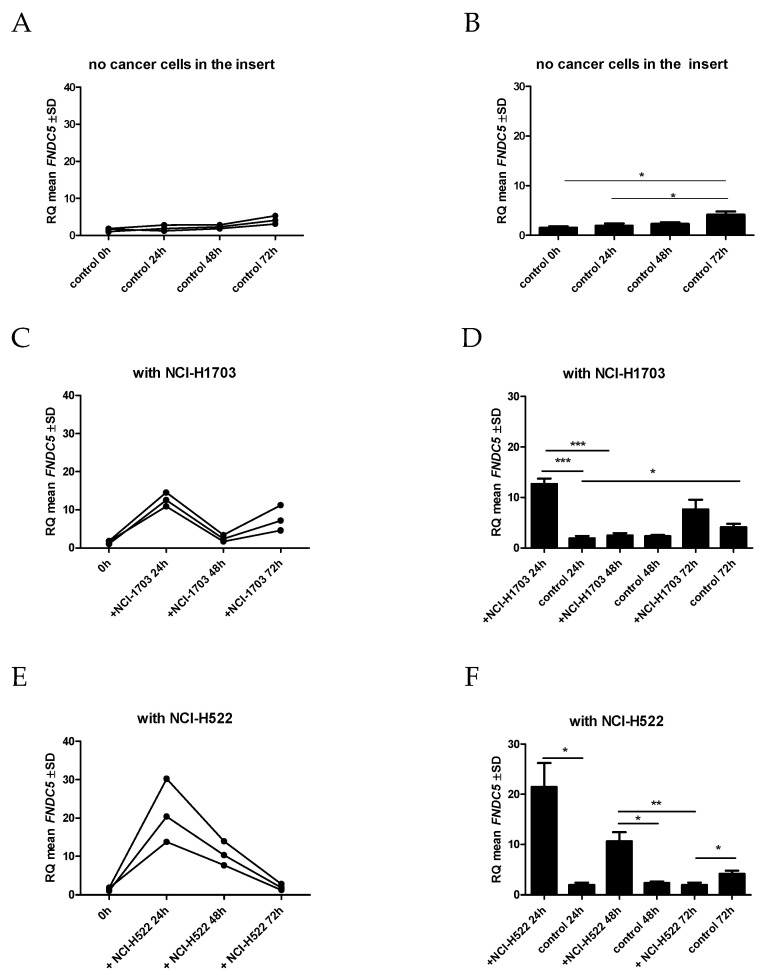
Comparison of the expression level of *FNDC5* mRNA after co-culture in IMR-90 cells in the empty insert (control) (**A**,**B**) and the insert with lung cancer cells [NCI-H1703 (**C**,**D**) and NCI-H522 (**E**,**F**)], * *p* ≤ 0.05, ** *p* ≤ 0.005, *** *p* < 0.001.

The effect of NCI-H1703 and NCI-H522 lung cancer cell lines on the *FNDC5* mRNA expression levels in normal fibroblasts of the IMR-90 line was investigated. A slight increase in the expression level of the *FNDC5* gene was observed in IMR-90 lung fibroblast cells placed in a 6-well plate with an empty insert (control). The difference was significantly higher only after 72 h, as compared to the expression of *FNDC5* mRNA after 24 h (Figure 10A,B).

On the other hand, the expression of *FNDC5* mRNA in IMR-90 cells was significantly higher after 24 h of co-culture when the cells of NCI-H1703 (*p* = 0.0007) (Figure 10C,D) or NCI-H522 (*p* = 0.0153) (Figure 9E,F) lines were added to the insert. The highest increase in *FNDC5* gene expression in IMR-90 cells was observed when NCI-H1703 cells, which are equivalent to lung squamous cell carcinoma, were added to the insert.

Moreover, within the next 72 h of the co-culture, the level of *FNDC5* mRNA increased again in the case of the co-culture with NCI-H1703. However, the level of *FNDC5* mRNA in IMR-90 cells after 72 h of co-culture with lung cancer cells of the NCI-H522 line was significantly lower than in control cells cultured with an empty insert (*p* = 0.0462).

### 2.9. Influence of Lung Cancer Cells on ESRRA mRNA Expression Levels in an In Vitro Model

The expression levels of the *ESRRA* gene in IMR-90 cells after co-culture with cells of the lung cancer line were also analyzed compared to the control. We noticed a decrease in the *ESRRA* mRNA level after incubation of IMR-90 in 6-well plates after 24 h, 48 h and 72 h.

**Figure 11:**
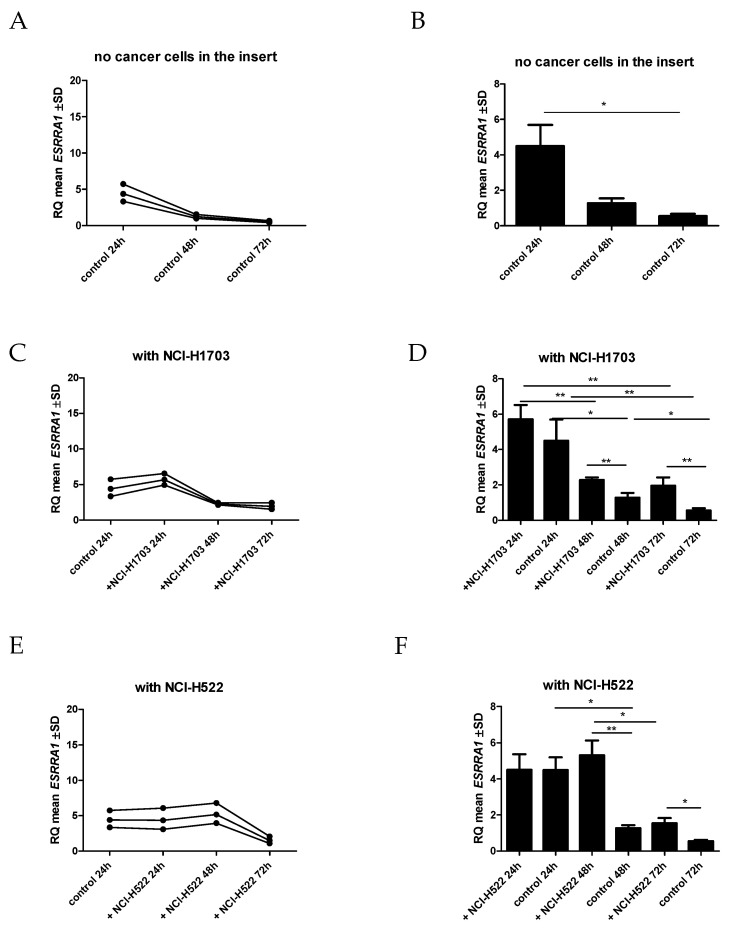
Comparison of the expression level of *ESRRA* mRNA after co-culture in IMR-90 cells in the empty insert (control) (**A**,**B**) and the insert with lung cancer cells [NCI-H1703 (**C**,**D**) and NCI-H522 (**E**,**F**)], * *p* ≤ 0.05, ** *p* ≤ 0.005.

However, we observed a slight increase in *ESRRA* expression levels in IMR-90 when cells of NCI-H1703 or NCI-H522 were added to the insert in co-cultivation after 24 h. A significant increase in *ESRRA* gene expression was observed after 48 h of co-culture with NCI-H1703 (*p* = 0.0050) and NCI-H522 (*p* = 0.0087), and after 72 h of co-cultivation with NCI-H1703 (*p* = 0.0068) and NCI-H522 (*p* = 0.0278), compared to the expression levels from IMR-90 cultured with the empty insert (Figure 11).

### 2.10. Ultrastructural Expression of Irisin/FNDC5 in Lung Cancer Cells

**Figure 12:**
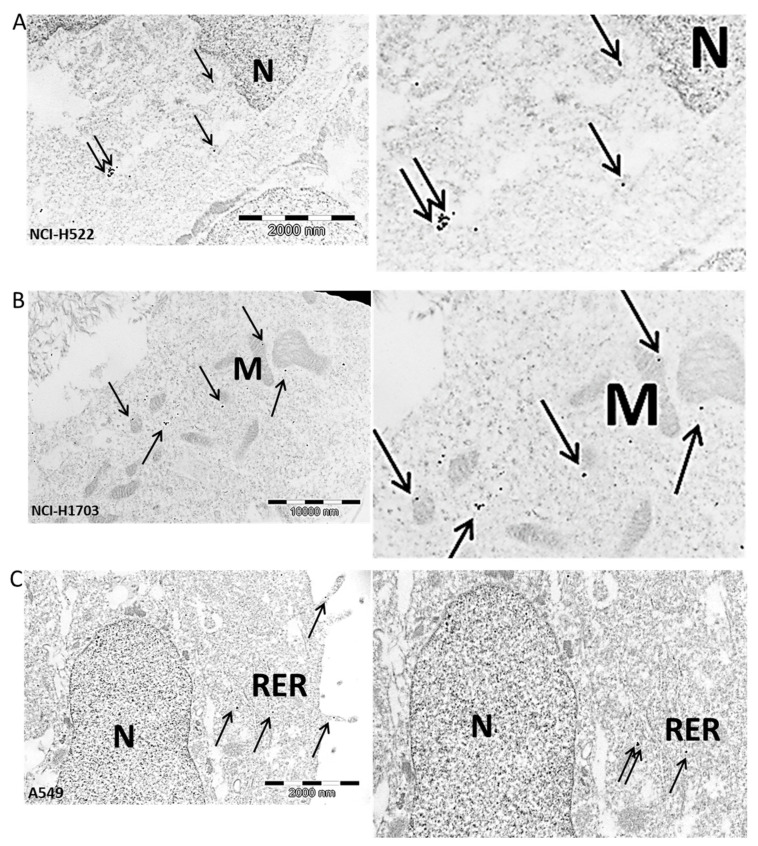
Positive immunogold reaction (black dots—indicated by arrows) point to irisin/FNDC5 expression in the cell cytoplasm in NCI-H522 cells—magnification on the right (**A**), in NCI-H1703 cell mitochondria-M membrane—magnification on the right (**B**), in rough endoplasmic reticulum-RER and in cytoplasmic extensions of A549 cell (**C**), *N*-nucleus, magnification ×25,000.

We also performed a study showing the expression of irisin/FNDC5 in NCI-H522, NCI-1703 and A549 lung cancer cells using the immunogold technique (Figure 12). The cells presented electron-light cytoplasm; a single elongated, euchromatic nucleus with sparse heterochromatin islets; and the irregular outline of the nuclear envelope. Large nucleoli had conspicuous granular components, a fibrillar center, and dense fibrillar components. The cell membrane created long cytoplasmatic protrusions. Abundant mitochondria with different shapes from elongated to round were unevenly distributed in the cytoplasm, with the inner membrane forming lamellar cristae. Cancer cells also contained anastomosing networks and interchanging channels of the rough endoplasmic reticulum, extensive arrays of microfilaments, and intermediate filaments which formed dense tufts. To improve the assessment of irisin/FNDC5 localization at the subcellular level, we enhanced the contrast of the phospholipid membrane and nucleic acids by post-staining with uranyl acetate and lead citrate, respectively. We demonstrated the presence of irisin in the cytoplasm, mitochondria, and rough endoplasmic reticulum of lung cancer cells. Moreover, we noted the secretion of irisin/FNDC5 from lung cancer cells into the extracellular space.

## 3. Discussion

In our study, we demonstrated a weak correlation between PGC-1α and irisin. Böstrom et al. [7] indicated that irisin expression was controlled by the transcription factor PGC-1α. The weak correlation between PGC-1α and irisin that we observed is surprising. Moreover, there have been no studies that showed a strong correlation between these proteins. This may suggest the existence of other factors that may have an additional impact on irisin expression in cancer cells and stromal cells of NSCLC. Perhaps, the weak correlation between PGC-1α and irisin is also due to other factors that should be investigated in the future.

In our investigation, we also observed a positive moderate correlation between irisin and ERRα in NSCLC stromal cells. Additionally, we found an association between the levels of *FNDC5* and *ESRRA* expression in NSCLC. In our previous study, we reported a high level of irisin expression in NSCLC stromal cells. Higher irisin expression in NSCLC stroma was a poor independent prognostic factor for patients. No correlations between irisin/FNDC5 and ERRα have been demonstrated yet in cancers or other diseases. However, Li et al. [30] observed that after ERRα knockdown of A549 cells their viability and migration potential were suppressed. The authors demonstrated higher expression levels of ERRα in more advanced stages of lung cancer (Stage III and IV). Moreover, they noted that patients with higher ERRα levels had shorter survival times. The explanation for the association between ERRα and irisin in NSCLC stromal cells that we observed may be in line with Wrann et al. [22]. They explored the influence of FNDC5 on brain-derived neurotrophic factor (BDNF) expression in the hippocampus. They observed that the ability of PGC-1α to induce *FNDC5* gene expression was dependent on ERRα and suggested that the PGC-1α/ERRα complex was essential for binding to the canonical estrogen response element (ERRE), which is close to the *FNDC5* gene location. PGC-1α cannot bind directly to DNA because it is a transcriptional co-activator. The correlation between irisin and ERRα that we observed suggests that there is a similar association in tumors. However, IHC only indicates a relationship and is not sufficient to prove it. Further studies are warranted to clarify the functional relationship of these proteins.

Overexpression of PGC-1α causes stimulation of ERRα expression [23]. Wrann et al. [22] observed that the knocking down of ERRα led to a lack of PGC-1α induction of the *FNDC5* gene in cultured cortical neurons. The relationship between irisin and ERRα may also be indicated by their participation in many metabolic processes. Each of them was suggested to influence glucose metabolism and oxidative metabolism in the mitochondria [5,8,10,18,30]. The role of ERRα and PGC-1α in NSCLCs has not been determined. However, studies have demonstrated their involvement in the cell cycle regulation and interactions between cells and the extracellular matrix. These observations indicate a possible involvement of ERRα and PGC-1α in regulating cancer cell proliferation as well as metastatic potential. The mechanism by which ERRα regulates NSCLC cell division and migration is not clear [35]. ERRα is involved in regulating c-Myc, p53, vascular endothelial growth factor (VEGF) and β-catenin expression. The PGC-1α is considered a surrogate ligand for ERRα, which participates in the regulation of mitochondrial genes, lipids and glucose homeostasis [5,35].

The presence of irisin in cancer cells and cancer stromal cells was described in our earlier paper [10]. In this study, we focused on the significance of the elevated irisin expression in stromal cells. One of the objectives was to verify whether the expression of *FNDC5* and *ESRRA* genes increased in stromal cells due to the presence of lung cancer cells. The conducted experiment in an in vitro model indicated that the presence of lung cancer cells could induce an increase in the expression of the *FNDC5* and *ESRRA* genes in stromal fibroblasts. In our study, we also investigated the influence of lung cancer cells on normal fibroblasts. Normal fibroblasts, when incubated with cells of various lung cancer lines, showed a significant increase in the *FNDC5* gene expression after only 24 h of incubation. This may confirm the expression of irisin/FNDC5 in tumor stromal cells found in our previous study [10]. In our former study, high levels of irisin/FNDC5 in stromal fibroblasts were an unfavorable prognostic factor associated with shorter patient survival.

The analysis of the results of *FNDC5* gene expression in normal fibroblasts after incubation with cancer cells may indicate that cancer cells could influence the alteration of gene expression levels in stromal cells and change their metabolism. Moreover, the association of *FNDC5* expression in stromal cells with PGC-1α and ERRα may indicate a potential role of irisin in reprogramming the metabolism of tumor stromal cells. We also noticed a change in *ESRRA* gene expression, which was also increased in normal fibroblasts after 48 h incubation with lung cancer cells. Similar studies using co-culture simulating tumor conditions were carried out by Yoriki et al. [36], who observed that induced overexpression of ERRα in endometrial cancer cells increased the expression of TGF-β and ERRα in stromal cells [36]. Correspondingly, earlier studies using co-culture also showed the presence of such a mechanism in breast cancer cells and their effects on stromal cells [37]. So far, many studies have demonstrated that cancer-associated fibroblasts (CAFs) are derived from normal fibroblasts in the tissue. Yoriki et al. [36] also observed an increased expression of the ERRα receptor as well as PGC-1α in normal endometrial fibroblasts (T-HESCs) after incubation with endometrial cancer cells. Their research is consistent with our findings. The alteration of *ESRRA* and *FNDC5* gene expression is perhaps associated with the activation of normal fibroblasts into CAFs. It might be the result of cross-talk between cancer cells and the neighboring cells. Yoriki et al. [36] also noted that silencing *ESRRA* expression inhibited factors associated with epithelial mesenchymal transition (EMT) in cancer cells and T-HESC cells. On the other hand, ERRα/PGC-1α overexpression increased the expression of EMT-related factors after exposure to TGF-β and decreased the level of E-cadherin. Rapid tumor growth can induce temporary malnutrition and hypoxia, which increase ERRα/PGC-1α expression in cancer cells. Both Yoriki et al. [36] and Matsushima et al. [38] found a relationship between ERRα/PGC-1α expression and promotion of tumor angiogenesis by inducing VEGF transcription. Therefore, ERRα could be associated with the process of angiogenesis and tumor cell invasion in the advanced stages of cancer [38]. This might also suggest the involvement of irisin in both processes.

Due to the changes in the cells after co-culture, we also checked the correlation of ERRα with clinicopathological factors, as well as diagnostic markers in NSCLC. We noticed high expressions of ERRα in NSCLC tissues, and its absence in NMLTs, which is consistent with the findings of studies on different types of cancer. The increase in ERRα expression was observed in breast [39], ovary [40], endometrium [38] and lung [30] cancer. In our study, a decrease in ERRα expression was noticeable with an increase in tumor size and the stage of the disease. These observations confirm the study findings of Li et al. [30], who demonstrated a positive effect of reduced ERRα expression on the proliferation, migration and invasion of lung cancer cells. We did not observe any relationship between the level of ERRα expression and the survival time of patients in the NSCLC group. However, in the case of the AC subtype, we observed an association of higher ERRα expression with longer overall survival times. On the other hand, Li et al. [30] indicated that higher expression in the AC subtype was associated with shorter survival time. However, Li et al. [30] investigated the relationship between the level of *ESRRA* expression and patients’ overall survival using the KM Plotter database in which mRNA expression was examined. In our study, we used IHC to detect the ERRα protein in NSCLC tumors. This may cause differences in the results. Moreover, this difference suggests the existence of additional epigenetic mechanisms influencing the formation of a functional protein of the ERRα. Moreover, Suzuki et al. [41] noted a correlation between the occurrence of higher ERRα expression and breast cancer (BC) recurrence. Patients with ERRα expression had shorter disease-free survival (DFS). They also showed that the presence of increased ERRα expression was an independent negative prognostic factor for the survival of patients with BC [41].

We observed ultrastructural irisin/FNDC5 expression in the cytoplasm of lung cancer cells, in mitochondria and in the rough reticulum. Furthermore, we also found that irisin was secreted outside the cells. The study of irisin localization in the ultrastructure of lung cancer cells using the immunogold technique confirmed our earlier observations using confocal and optical microscopy [10]. In our previous study, we detected irisin/FNDC5 expression in the cytoplasm of lung cancer cells. Additionally, the ultrastructural expression of irisin/FNDC5 in the mitochondria is consistent with their function in the conversion of white adipose tissue (WAT) to brown adipose tissue (BAT) by increasing UCP1 expression [7].

The limitation of our study was the use of TMAs, which indicated the expression level of the investigated proteins in the part of whole sections. The comparative studies performed by our team showed that the results obtained with the TMAs adequately reflect the findings obtained from the entire section.

To conclude, next to PGC-1α, the ERRα receptor may be an additional factor that participates in the control of irisin expression in lung cancer cells. Moreover, normal fibroblasts revealed the upregulation of the *FNDC5* gene under the influence of lung cancer cells. However, more research is needed to determine the functional relationship between these proteins and to confirm their involvement in the control of *FNDC5* gene expression in NSCLC, as well as potentially other types of cancer. In addition, we observed the potential usefulness of ERRα expression in the assessment of clinicopathological parameters such as tumor size and NSCLC stage. Moreover, in the AC subtype, high ERRα expression was associated with longer patient survival. In the future, this receptor could be potentially used as a therapeutic target or a potential new diagnostic marker.

## 4. Materials and Methods

### 4.1. Patient Cohort

From 2007 to 2011, 1371 patients diagnosed with lung cancer underwent tumor resection at the Department of Thoracic Surgery at Wroclaw Medical University, Poland. Archival and frozen material of lung cancer specimens were obtained from patients. After selection, 860 patients were enrolled. Other patients were excluded due to prior chemotherapy or a tumor size too small to perform tissue microarrays (TMAs). The control group consisted of 140 non-malignant lung tissue (NMLT) sections. Fresh frozen NSCLC specimens (n = 56) and NMLTs (n = 16) were used for molecular studies. All patients gave their written informed consent. The study concept was approved by the Wroclaw Medical University Institutional Review Board and the Bioethics Committee (ID No. KB-83/2011; 3 March 2011 and KB-222/2020; 20 April 2020). Histopathological evaluation and pathological staging were performed according to the World Health Organization criteria. The archival material consisted of 860 cases of NSCLC, including ACs (n = 344), SCCs (n = 375), adenosquamous carcinomas (n = 32), and other and unclassified NSCLCs (n = 109). Clinicopathological characteristics of NSCLC patients are given in Table 1, Table 2 and Table 3.

### 4.2. Cell Culture Line and Cell Co-Culture

Co-culture and molecular biology studies were performed using the adherent lung cancer cell line NCI-H522 (equivalent to lung adenocarcinoma) and NCI-H1703 (equivalent to lung squamous cell carcinoma) from the American Type Culture Collection (Manassas, VA, USA). The normal lung fibroblast IMR-90 cell line was used as a cell substitute for normal cells surrounding lung tumor. A549 (equivalent to lung adenocarcinoma) from the American Type Culture Collection (Manassas, VA, USA), NCI-H522 and NCI-H1703 were used to perform immunogold reactions. NCI-H522 and NCI-H1703 cells were cultured in RPMI-1640 medium (Lonza, Basel, Switzerland). EMEM medium (Lonza, Basel, Switzerland) was used to culture IMR-90. F-12K medium (Lonza, Basel, Switzerland) was used to culture A549 cells. All media were supplemented with 10% of fetal bovine serum (FBS) (Merck, Darmstadt, Germany) and 1% of L-glutamine/penicillin/streptomycin (Merck, Darmstadt, Germany). Constant conditions of 37 °C, 5% CO_2_ concentration, and a 95% humidity level for cell cultures and co-cultures were maintained in the HERA (Heraeus, Hanau, Germany) cell incubator.

To prepare co-culture, cells of the normal lung fibroblast line IMR-90 were cultured in Thin Cert Cell Culture 6-well plates with a 0.4-µm diameter of pore size inserts (Greiner Bio-One, Kremsmünster, Austria) that prevented cell migration but allowed factor exchange. Two 10^5^ IMR-90 cells were seeded on each well. Moreover, five 10^4^ cells of specific lung cancer lines (i.e., NCI-H1703 and NCI-H522) were placed on the inserts. As a control, IMR-90 cells were grown in the insert with the culture medium only, without lung cancer cells. The cultures were conducted for 24 h, 48 h and 72 h. Next, IMR-90 cell pellets were collected, from which total mRNA was isolated and used for further molecular studies. The co-cultures were repeated three times for each time period.

### 4.3. Immunohistochemical (IHC) Reactions on Tissue Microarrays (TMAs)

Tissue microarrays (TMAs) were performed on 860 NSCLC and 140 control sections. The slides with the whole NSCLC tissues or control tissue sections were hematoxylin- and eosin-stained and scanned with the use of the Pannoramic Midi II (3D HISTECH Ltd., Budapest, Hungary) histological scanner. The research pathologists selected three demonstrative sites with cancer using the Pannoramic Viewer (3D HISTECH Ltd., Budapest, Hungary) Software. The selected cancer sites were transferred to the tissue arrays using the TMA Grand Master (3D HISTECH Ltd., Budapest, Hungary). The core of the transferred sites was 1.5 mm. Immunohistochemical (IHC) reactions were performed on 4-µm TMA sections with NSCLC and the control. Deparaffinization, hydration, and thermal epitope demasking were performed in a low pH Target Retrieval Solution (Agilent Technologies, Santa Clara, CA, USA) for 20 min at 97 °C in a Dako PT Link (Dako, Glostrup, Denmark) apparatus. Antigen expressions were detected using specific primary antibodies: polyclonal rabbit anti-irisin/FNDC5 (1:50 dilution; code no. NBP2-14024; Novus Biologicals, Littleton, CO, USA), polyclonal rabbit anti-ERRα (1:100 dilution, code GTX108166; GeneTex, Irvine, CA, USA), polyclonal rabbit anti-PGC-1α (1:3200 dilution, code NBP1-04676; Novus Biologicals, Littleton, CO, USA), monoclonal mouse anti-PD-L1 (ready-to-use, Clone DAKO-p63, code IR662; Dako, Glostrup, Denmark), polyclonal rabbit anti-EGFR (1:100 dilution, code HPA018530, Sigma, Munich, Germany), monoclonal mouse anti-TTF-1 (ready to use, clone 8G7G3/1, code IR056; Dako, Glostrup, Denmark) and monoclonal mouse anti-p63 (ready-to-use, clone DAKO-p63, code IR662; Dako, Glostrup, Denmark). Secondary goat anti-rabbit immunoglobulin antibodies (EnVision/HRP; Dako, Glostrup, Denmark) and anti-mouse immunoglobulin antibodies (EnVision/HRP; Dako, Glostrup, Denmark) were coupled to a dextran core linked to peroxidase. The color reaction was obtained using 3,3′-diaminobenzidine tetrachlorohydrate. Additionally, TMA sections were stained with hematoxylin (EnVisionTM FLEX Hematoxylin; Dako, Glostrup, Denmark). To visualize the antigens, a DAKO Autostainer Link48 (Dako, Glostrup, Denmark) automated system and an EnVision FLEX kit (Dako, Glostrup, Denmark) were used according to the manufacturer’s instructions. Negative control was made without a primary antibody.

### 4.4. Immunohistochemistry (IHC) Evaluation

The slides with IHC staining were estimated by two independent research pathologists (PD and KN). The assessment was carried out using a BX41 (Olympus, Tokyo, Japan) light microscope coupled with a visual circuit and the Cell D (Olympus, Tokyo, Japan) software. The evaluation of IHC staining was performed at ×200 magnification.

The expression levels of ERRα, TTF-1 and p63 were determined using the five-point evaluation scale (0—no expression, 1 point—>0–10%, 2 points—>10–25%, 3 points—>25–50%, 4 points—>50%). The semiquantitative method immunoreactive score (IRS) according to Remmele and Stegner was used for the evaluation of the cytoplasmic and membranous expression of irisin and PGC-1α (in the cell and the stroma of NSCLC) and EGFR [42]. The final result was the product of the multiplication of the points from the percentage of positive cancer cells (0 points—lack of expression, 1 point—>1–10%, 2 points—>10–50%, 3 points—>50–80%, 4 points—>80%) and the intensity of the color reaction (1—weak, 2—moderate, 3—strong). To evaluate PD-L1 expression, the Tumor Proportion Score (TPS) that is applied routinely in diagnostic settings was used (0 point < 1%, 1 point ≥ 1% to <50% and 2 points ≥ 50%) [2]. Tumor cells with a positive cytoplasmic expression were excluded.

### 4.5. Real-Time PCR (RT-PCR)

Real-time PCR (RT-PCR) reactions were performed on 56 fresh-frozen NSCLCs and 16 normal lung tissues. Additionally, RT-PCR was made to examine *FNDC5* and *ESRRA* mRNA in IMR-90 cells from co-culture and control culture without lung cancer cells. The RNeasy Mini Kit (Qiagen, Venelo, The Netherlands) was used for RNA isolation. The High-Capacity cDNA Reverse Transcription Kit (Applied Biosystems, Waltham, MA, USA) and RNase Inhibitor (Applied Biosystems, Waltham, MA, USA) were used to perform the reverse transcription reaction. The 7900HT Fast (Applied Biosystems, Waltham, MA, USA) Real-Time PCR System and the relative quantification (RQ) method were used to analyze the FNDC5 mRNA expression (FNDC5; Assay ID: Hs00401006_m1, TaqMan Gene Expression Assay, Applied Biosystems, Waltham, MA, USA) and ESRRA mRNA expression (ESRRA; Assay ID: Hs00607062_gH, TaqMan Gene Expression Assay, Applied Biosystems, Waltham, MA, USA) in cell lines and tissues. The RQ Manager 1.2 (Applied Biosystems, Waltham, MA, USA) software was used to perform the analysis. The results were standardized and evaluated according to the reference gene of β-actin expression (ACTB; TaqMan Gene Expression Assay, Applied Biosystems, Waltham, MA, USA). The RT-PCR analysis was performed in three repetitions.

### 4.6. Transmission Electron Microscopy (TEM) Procedure

NCI-H522, NCI-H1703 and A549 cells were fixed in a freshly made 4% formaldehyde solution (25 min RT) diluted in phosphate buffer saline (PBS, pH 7.4). After fixation, the cells were scraped out with a sterile spatula and the cell suspensions were centrifuged 3 times at 1800 rpm for 8 min, then the fixative was rinsed with PBS and distilled water. Next, the droplets of bovine thrombin (1 amp. with 400 a.u. lyophilized dissolved in 5 mL of PBS Biomed, Lublin, Poland) and fibrinogen (1 mg/mL; Merck KGaA, Darmstadt, Germany) were placed into Falcon tubes with the cell pellets. The contents were gently shaken until the cells were entrapped within the fibrin clot. Next, the cell clots were post-fixed for 7 min in 0.25% (*w*/*v*) osmium tetroxide diluted in PBS (Serva Electrophoresis, Heidelberg, Germany). The time for OsO4 post-fixation was carefully controlled and after 7 min the fixative was washed with PBS (3 × 5 min). In the next step, the specimens were passed through a series of increasingly concentrated ethanol solutions (Stanlab, Lublin, Poland) for 10 min per each step at RT and left overnight at 4 °C in 70%. Afterwards, the samples were incubated with a mixture of 99.8% ethanol and LR White resin (LR White Embedding Media, Medium catalyzed, Polysciences, Inc., Warrington, PA, USA, cat. # 17411M-500) in the following proportions: 2:1 (20 min), 1:1 (1 h), and 1:2 (1 h), respectively. Then, the samples were placed in pure resin and incubated overnight at 4 °C. Finally, the resin-saturated material was transferred to specimen boxes (flat embedding molds, Pelco, Ted Pella, Redding, CA, USA) and deluged with pure LR White resin. Polymerization of the LR White resin blocks was performed at 55 °C for 48 h.

Next, LR White blocks were trimmed and cut on the ultramicrotome Power Tome XL (RMC, Tucson, AZ, USA) with a histo-diamond knife (Diatome, Nidau, Switzerland) into semithin 600-nm-thick sections, which were stained with toluidine blue (Serva Electrophoresis, Heidelberg, Germany) and closed with a Euparal mounting agent (Carl Roth, Mannheim, Germany).

For immunolocalization of irisin, a culture A549 cell line was carefully selected for making ultrathin 70-nm-thick sections using an ultra 45° diamond knife (Diatome, Nidau, Switzerland). The ultrathin sections were transferred to the dull side of the TEM nickel grids (200 mesh, Ted Pella, Redding, CA, USA). During the whole procedure, the grids were incubated on the top of the droplet of the appropriate reagents with the ultrathin sections face down at RT. The ultrathin sections were incubated in fresh glycine 0.02 M (Biotechnology grade, BioShop Canada Inc., Burlington, ON, Canada) dissolved in PBS (1 time for 10 min) to quench free aldehyde groups, followed by gentle rinsing with PBS (1X). Then, for membrane permeabilization, the grids were incubated with 0.1% Triton X-100 in PBS (Reagent grade, Bioshop, Canada Inc., Burlington, ON, Canada, cat. # TRX 506.500), diluted in PBS 2 times for 5 min., followed by washing the grids for 5 min. with PBS (3X). Non-specific antigen-binding sites were blocked for 1 h in a solution of 1% bovine serum albumin (BSA, albumin fraction V, Carl Roth, Mannheim, Germany) and rinsed with PBS for 5 min. Afterwards, the grids were transferred to the surface of a 30 µL droplet of the polyclonal rabbit anti-irisin/FNDC5 antibody (1:10 dilution, code no. NBP2-14024; Novus Biologicals, Littleton, CO, USA) diluted in 0.1% BSA in PBS, then the sections were washed in PBS. Subsequently, the samples were labeled with the secondary antibody conjugated with colloidal gold particles (1:10 dilution, code no. ab27237Abcam, Cambridge, UK, Goat Anti-Rabbit IgG H&L, 20 nm Gold, preabsorbed) prepared in 0.1% BSA in PBS for 1 h at RT (dark chamber). The following step was rinsing the grids in PBS and in distilled water. To preserve the ultrastructure of the cell membranes, the sections were fixed in 1% glutaraldehyde (Serva Electrophoresis, Heidelberg, Germany) diluted in PBS for 5 min; the fixative was rinsed with distilled water. The sections underwent double counterstaining with uranyl acetate (10 min) and lead citrate trihydrate (5 min) (Serva, Electrophoresis, Heidelberg, Germany), and then were rinsed with distilled water. The samples were visualized under TEM JEM-1011 (Jeol, Tokyo, Japan) operating at the accelerating voltage of 80 kV. Electron microphotographs were obtained using the TEM imaging platform iTEM1233, equipped with a Morada Camera (Olympus, Tokyo, Japan) at magnification ranging from 5 to 20 K.

## Figures and Tables

**Table 1:** Clinicopathological characteristics of patients with non-small cell lung cancer (NSCLC) related to low and high expression of irisin/FNDC5 (Chi^2^ test analysis), significance in bold.

Clinicopathological Parameter	n 860 (%)	Irisin/FNDC5 Expression in NSCLC Cancer Cells			Irisin/FNDC5 Expression in NSCLC Stromal Cells		
		Low ≤1.0	High >1.0	Chi^2^ Test *p* Value	Low ≤4.0	High >4.0	Chi^2^ Test *p* Value
**Age** **≤60** **>60**	354 (41.2) 506 (58.8)	194 (54.8) 258 (51)	159 (45.2) 249 (49)	0.2397	197 (55.6) 265 (52.4)	156 (44.3) 242 (47.6)	0.3059
**Sex** **Male** **Female**	636 (74) 224 (26)	353 (55.5) 99 (44.2)	282 (44.5) 126 (55.8)	**0.0028**	326 (51.2) 136 (60.7)	309 (48.8) 89 (39.3)	**0.0186**
**Histological subtype** **AC** **SCC** **Adenosquamous** **other**	344 (40) 375 (43.6) 32 (3.7) 109 (12.7)	140 (40.7) 233 (62.1) 21 (65.6) 59 (54.1)	204 (59.3) 142 (37.8) 11 (34.4) 50 (45.9)	**<0.0001**	220 (63.9) 161 (42.9) 13 (40.6) 68 (62.4)	124 (36.1) 214 (57.1) 19 (59.4) 41 (37.6)	**<0.0001**
**Tumor size (T)** **T1-T2** **T3-T4**	584 (67.9) 276 (32.1)	291 (49.8) 161 (58.3)	297 (50.2) 111 (41.7)	**0.0081**	314 (53.8) 148 (53.6)	273 (46.2) 125 (46.4)	0.8437
**Lymph nodes (N)** **N0** **N1** **N2-N3**	573 (66.5) 151 (17.5) 136 (16)	297 (51.8) 88 (58.3) 67 (49.3)	275 (48.2) 63 (41.7) 70 (50.7)	0.2457	306 (53.4) 73 (48.3) 83 (61)	266 (46.6) 78 (51.7) 54 (39)	0.1129
**Stage** **I** **II** **III-IV**	314 (36.5) 291 (33.8) 255 (29.7)	151 (48.1) 159 (54.6) 142 (55.7)	162 (51.9) 132 (45.4) 114 (44.3)	0.1562	176 (56) 138 (53.3) 148 (48.5)	137 (44) 153 (46.7) 108 (51.5)	**0.0144**
**Grade of malignancy (G)** **G1** **G2** **G3**	83 (9.6) 631 (73.4) 146 (17)	35 (42.2) 329 (52.1) 90 (61.6)	48 (57.8) 302 (47.9) 56 (38.4)	**0.0146**	51 (61.4) 322 (51) 89 (62.2)	32 (38.6) 309 (49) 57 (37.8)	**0.0316**

**Table 2:** Clinicopathological characteristics of patients with non-small cell lung cancer (NSCLC) related to low and high expression of ERRα (Chi^2^ test analysis), significance in bold.

Clinicopathological Parameter	n 860 (%)	ERRα Expression in NSCLC Cancer Cells		
		Low ≤2.6	High >2.6	Chi^2^ Test *p* Value
**Age**				0.9534
**≤60**	354 (41.2)	198 (55.9)	156 (44.1)	
**>60**	506 (58.8)	282 (55.7)	224 (44.3)	
**Sex**				0.0722
**Male**	636 (74)	294 (46.2)	342 (53.8)	
**Female**	224 (26)	88 (39.3)	136 (60.7)	
**Histological subtype**				**<0.0001**
**AC**	344 (40)	237 (68.9)	107 (31.1)	
**SCC**	375 (43.6)	163 (43.5)	212 (56.5)	
**Adenosquamous**	32 (3.7)	16 (50)	16 (50)	
**other**	109 (12.7)	63 (58)	46 (42)	
**Tumor size (T)**				0.0987
**T1-T2**	584 (67.9)	312 (53.4)	272 (46.6)	
**T3-4**	276 (32.1)	164 (59.4)	112 (40.6)	
**Lymph nodes (N)**				**0.0196**
**N0**	573 (66.5)	311 (54.3)	262 (45.7)	
**N1**	151 (17.5)	77 (51)	74 (49)	
**N2-N3**	136 (16)	90 (66.2)	46 (33.8)	
**Stage**				**0.0119**
**I**	314 (36.5)	158 (50.3)	156 (49.7)	
**II**	291 (33.8)	160 (55)	131 (45)	
**III-IV**	255 (29.7)	160 (62.7)	95 (37.3)	
**Grade of malignancy**				0.5181
**G1**	83 (9.6)	51 (61)	32 (39)	
**G2**	631 (73.4)	346 (54.8)	285 (45.2)	
**G3**	146 (17)	82 (56.2)	64 (43.8)	

**Table 3:** Clinicopathological characteristics of patients with non-small cell lung cancer (NSCLC) related to low and high expression of PGC-1α (Chi^2^ test analysis), significance in bold.

Clinicopathological Parameter	n 860 (%)	PGC-1α Expression in NSCLC Cancer Cells			PGC1α Expression in NSCLC Stromal Cells		
		Low ≤4.6	High >4.6	Chi^2^ Test *p* Value	Low ≤4.5	High >4.5	Chi^2^ Test *p* Value
**Age** **≤60** **>60**	354 (41.2) 506 (58.8)	192 (54.2) 288 (56.9)	162 (45.8) 218 (43.1)	0.4361	200 (56.5) 278 (54.9)	154 (43.5) 228 (45.1)	0.6512
**Sex** **Male** **Female**	636 (74) 224 (26)	350 (55) 130 (58)	286 (45) 94 (42)	0.4362	406 (63.8) 72 (32.1)	230 (36.2) 152 (67.9)	**<0.0001**
**Histological subtype** **AC** **SCC** **Adenosquamous** **other**	344 (40) 375 (43.6) 32 (3.7) 109 (12.7)	234 (68) 199 (53) 19 (59) 28 (25.7)	110 (32) 176 (47) 13 (41) 81 (74.3)	**<0.0001**	247 (71.8) 175 (46.6) 15 (46.9) 41 (37.6)	97 (28.2) 200 (53.3) 17 (53.1) 68 (62.4)	**<0.0001**
**Tumor size (T)** **T1-T2** **T3-T4**	584 (67.9) 276 (32.1)	323 (55.3) 99 (35.9)	261 (44.7) 177 (64.1)	**<0.0001**	324 (55.5) 154 (55.8)	260 (44.5) 122 (44.2)	0.9303
**Lymph nodes (N)** **N0** **N1** **N2-N3**	573 (66.5) 151 (17.5) 136 (16)	315 (55) 90 (60) 75 (55)	258 (45) 61 (40) 61 (45)	0.5864	311 (54.3) 82 (54.3) 85 (62.5)	262 (45.7) 69 (45.7) 51 (37.5)	0.2089
**Stage** **I** **II** **III-IV**	314 (36.5) 291 (33.8) 255 (29.7)	92 (29.3) 161 (55.2) 227 (89)	222 (70.7) 130 (44.8) 28 (11)	**<0.0001**	171 (54.4) 155 (53.3) 124 (48.5)	143 (45.6) 136 (46.7) 131 (51.5)	0.3546
**Grade of malignancy (G)** **G1** **G2** **G3**	83 (9.6) 631 (73.4) 146 (20.3)	50 (60) 369 (58.5) 50 (34.2)	23 (40) 262 (41.5) 96 (65.8)	**<0.0001**	16 (20) 406 (64.3) 71 (48.5)	67 (80) 225 (35.7) 75 (51.5)	**0.0004**

## Data Availability

The raw data will be made available upon reasonable request. To access protocols or datasets contact katarzyna.nowinska@umw.edu.pl.

## Abbreviations

FNDC5—fibronectin type III domain-containing protein 5, WAT—white adipose tissue, BAT—brown adipose tissue, UCP1—uncoupling protein 1, PPAR—peroxisome proliferator-activated receptor, PGC1α—peroxisome proliferator-activated receptor gamma coactivator 1 alpha, CAFs—cancer-associated fibroblasts, VEGF—vascular endothelial growth factor, EGFR—epidermal growth factor receptor, ALK—anaplastic lymphoma kinase, KRAS—Kirsten rat sarcoma viral oncogene homologue, AMPK—5′AMP-activated protein kinase, MAPK—Mitogen-activated protein kinase, EMT—epithelial–mesenchymal transition, NSCLC—non-small-cell lung carcinoma, NMLT—non-malignant lung tissues, AC—lung adenocarcinoma, SCC—lung squamous cell cancer, ERRα—estrogen related receptor α, ERRE—estrogen response elements, PD-L1—programmed death ligand 1, PD-1—programmed death ligand 1 receptor, TTF-1—thyroid transcription factor 1, BDNF—brain-derived neurotrophic factor, TEM—transmission electron microscopy, TMA—tissue microarrays, PBS—phosphate buffered saline, BSA—bovine serum albumin, IHC—immunohistochemistry, RT-PCR—real-time PCR, RQ—relative quantification, OS—overall survival, DFS—disease-free survival, BC—breast cancer, T—tumor size, G—grade of malignancy, N—lymph node.
